# Hepatitis C and hepatitis B seroprevalence and associated risk factors in hemodialysis patients in Guilan province, north of Iran: HCV and HBV seroprevalence in hemodialysis patients

**Published:** 2011-03-01

**Authors:** Farahnaz Joukar, Sepiedeh Besharati, Hasan Mirpour, Fariborz Mansour-Ghanaei

**Affiliations:** 1 Gastrointestinal and Liver Diseases Research Center, Guilan University of Medical Sciences, Rasht, IR Iran

**Keywords:** Hepatitis B, Hepatitis C, Hemodialysis

## Abstract

**Background:**

Hepatitis C virus (HCV) and hepatitis B virus (HBV) infection are especially problematic in patients with end-stage renal disease who are undergoing hemodialysis (HD).

**Objectives:**

To determine the prevalence of HCV and HBV infection in HD population in Guilan, north of Iran.

**Patients and Methods:**

In a cross-sectional study, from May to September 2009, in 11 different hemodialysis units in Guilan province, North of Iran, clinical data such as age, gender, duration of dialysis, HBsAg and anti-HCV antibody of 514 HD patients were recorded. Patients with positive antibodies against HCV were tested for HCV RNA.

**Results:**

From 514 patients, 286 (55.64%) were male. 61 (11.9%) patients were anti-HCV-positive and 31 (50.8%) were HCV PCR-positive. There was significant relationship between HCV Ab-positivity with gender and HD duration (p < 0.05). There was significant difference between the mean HD duration in anti-HCV-positive and anti-HCV-negative patients (p < 0.05). Also, significant relationship was found between HCV RNA-positivity with gender and HD duration (p < 0.05). Seven (1.4%) patients were positive for HBsAg. Two (0.38 %) were found positive for both HBsAg and anti-HCV antibody.

**Conclusions:**

There is low a prevalence of HCV and HBV in HD patients in our region. The rate can be decreased by HBV vaccination of end-stage renal disease patients before setting chronic HD, antiviral treatment and isolation of infected individuals.

## Background

Hepatitis C virus (HCV) infection is especially problematic in patients with end-stage renal disease (ESRD) who are undergoing hemodialysis (HD) [1]. The prevalence of HCV infection is higher among HD patients than in the general population, and several routes of transmission are thought to originate from the HD unit [1][2]. There is wide variation in the prevalence of HCV infection among different HD units and countries as shown by Dialysis Outcomes and Practice Patterns Study (DOPPS). The mean prevalence of HCV in different HD facilities is 13.5% and varied among countries from 2.6% to 22.9% [3]. The main reasons for such a high incidence of infections are a high prevalence of HCV infection in the general population, lack of standard infection precautions and effective vaccination, inadequate disinfection procedures of dialysis machines and other medical equipment, as well as spread of infection from patient to patient, especially in dialysis centers with a high percentage of infected patients. The diagnostic procedures used in the evaluation of HCV infection are detection of anti-HCV antibodies, identification of HCV RNA, counts of virus copies, and identification of its genome [4][5]. HCV infection is associated with greater mortality [6][7][8]. As a cause of death, hepatocellular carcinoma and liver cirrhosis are significantly more frequent among anti-HCV-positive than anti-HCV-negative dialysis patients [9]. Risk factors for HCV infection in dialysis patients include number of blood transfusions, duration of HD, mode of dialysis, prevalence of HCV infection in the dialysis unit, previous organ transplantation, intravenous drug use, male gender, older age, previous HBV infection and nosocomial transmission of HCV in HD units [10][11][12][13].

HBV infection is less prevalent than HCV in HD units. The rate of serum HBsAg seropositivity on maintenance HD in the developed world is currently low (< 10%) but outbreaks of acute HBV infection continue to occur in this setting. The prevalence of HBV infection within dialysis units in developing countries however is higher (2%-20%), according to several reports [14]. HBV infection is a major clinical problem, as it can lead to many serious consequences, including acute and chronic hepatitis, cirrhosis, hepatocellular carcinoma and hepatic failure [15][16]. Universal precaution measures should be strictly observed and the segregation of HBsAg-positive patients on HD should be practiced. Early vaccination against HBV before the start of ESRD remains the best way to secure immunological protection against HBV infection in dialysis patients [17].

## Objectives

In this study we aimed to determine evaluation of HCV and HBV infection in HD population in Guilan, North of Iran.

## Patients and Methods

In a cross-sectional study, clinical and epidemiological data were obtained from May to September 2009 in 11 different HD units in Guilan province, northern Iran A total of 514 patients were recruited from different cities of Anzali (n = 34), Astaneh (n = 36), Astara (n = 20), Fouman (n = 18), Lahijan (n = 56), Langaroud (n = 38), Rasht (n = 159), Roudbar (n = 17), Roudsar (n = 57), Soumesara (n = 40), and Talesh (n = 39). Clinical data such as age, gender, duration of dialysis, HBsAg and anti-HCV antibody status were recorded. Serological testing for HBsAg and anti-HCV antibody were performed on the recruited HD patients using third generation enzyme-linked immunosorbent assay (ELISA) kit. Consecutively, patients with positive antibodies against HCV were tested by seroconversion and real-time polymerase chain reaction (PCR) for detecting HCV RNA to eliminate false-negative cases. Based on the duration of HD, patients were divided into four groups of < 5, 5-10, 10-15, and > 15 years.

SPSS® ver 14 for Windows® was used to analyze data. Quantitative variables were expressed as mean ± SD. To evaluate risk factors associated with HCV infection, x(2) test was used. Student's t test was used to compare between means of two continuous variables. A p value < 0.05 was considered statistically significant.

## Results

Of 514 patients studied, 228 (44.35%) were female and 286 (55.64%) were male (Table 1). The mean age of participants was 54.8 (range: 16-66) years. In this study, 61 (11.9%) were anti-HCV-positive and 31 (50.8%) were HCV PCR-positive. Among those with positive anti-HCV antibody, 26 aged between 50 and 70 years. There was significant relationship between HCV antibody positivity with gender and dialysis duration (p < 0.05). No correlation was found between age with HCV antibody positivity (Table 1). The mean ± SD dialysis duration in anti-HCV-positive and anti-HCV-negative patients was 5.47 ± 4.62 and 3.10 ± 2.75 years, respectively (p < 0.05). Significant correlation was found between HCV RNA-positivity with gender and dialysis duration (p < 0.05). On the contrary, no significant correlation was found between age and HCV RNA-positivity (Table 1). The mean ± SD dialysis duration in HCV RNA-positive and HCV RNA-negative patients was 6.15±5.08 and 4.72 ± 4.02 years, respectively (p > 0.05). In this study, seven (1.4%) patients were HBsAg positive, six (1.16%) HBsAg-positive patients were male, four (0.7%) aged 50-70 years, and five (0.9%) had dialysis duration of < 5 years (Table 1). Two (0.38 %) patients were found positive for both HBsAg and anti-HCV antibody.

**Table 1:** HCV antibody, HCV RNA and HBsAgdistribution according to gender, age group, dialysis duration in hemodialysis patients

	**HCV Antibody**				**HCV RNA**				**HBsAg**		
	**Positive **No (%)	**Negative **No (%)	**Total**	**p-value**	**Positive **No (%)	**Negative **No (%)	**Total**	**p-value**	**Positive **No (%)	**Negative **No (%)	**Total**
**Gender**				<0.05				<0.05			
**Male**	41 (14.3)	245 (85.7)	286		41 (14.3)	245 (85.7)	286		6 (2.1)	280 (97.9)	286
**Female**	20 (8.8)	208 (91.2)	228		20 (8.8)	208 (91.2)	228		1 (0.4)	227 (99.6)	228
**Age** (year)				NS				NS			
**<30**	7 (16)	39 (84)	46		7 (16)	39 (84)	46		0 (0)	46 (100)	46
**30–50**	21 (17.8)	97 (82.2)	118		21 (17.8)	97 (82.2)	118		2 (1.7)	116 (98.3)	118
**50–70**	26 (10.4)	224 (89.6)	250		26 (10.4)	224 (89.6)	250		4 (1.6)	246 (98.4)	250
**>70**	7 (7)	93 (93)	100		7 (7)	93 (93)	100		1 (1.1)	99 (99)	100
**Dialysis Duration** (year)				<0.05				<0.05			
**< 5**	38 (9)	382 (91)	420		38 (9)	382 (91)	420		5 (1.2)	415 (98.8)	420
**5–10**	15 (20)	61 (80)	76		15 (20)	61 (80)	76		2 (3)	74 (97)	76
**10–15**	5 (38)	8 (62)	13		5 (38)	8 (62)	13		0 (0.0)	13 (100)	13
**> 15**	3 (60)	2 (40)	5		3 (60)	2 (40)	5		0 (0.0)	5 (100)	5

## Discussion

HCV infection remains highly prevalent both in developed [18] and less-developed countries [19]. In spite of considerable decline in the incidence and prevalence of HCV infection among HD patients in many countries, this infection still remains a major problem among patients on maintenance HD [20][21]. The prevalence of HCV infection found in our HD patients (11.9%) was lower than that seen in other studies. Telaku, et al, studied 583 ESRD patients on maintenance HD from six HD centers in Kosova. They reported an anti-HCV antibody prevalence rate of 43% [14]. In a descriptive study conducted by Alavian, et al, the prevalence of positive HCV antibodies decreased from 14.4% in 1999 to 4.5% in 2006 [22]. Assarehzadegan, et al, studied 214 HD patients in West-south of Iran and found 34 patients positive for anti-HCV (7.9%, 95% CI: 4.32%-11.56%). In that study, although anti-HCV positivity in HD patients in Khuzestan province was smaller than those found in some other Iranian provinces and neighboring countries, the prevalence was still high [23]. A retrospective study performed by Boulaajaj, et al, on 186 chronic HD patients, reported a high prevalence of HCV infection (76%) [24]. Olut, et al, showed that seroprevalence of HCV infection was 19% among 437 HD patients [14][15][16][17][18][19][20][21][22][23][24][25]. Silva, et al reported that anti-HCV seroprevalence among HD patients was 10.5% [26]. Mansour-Ghanaei, et al, showed that 18.40% of 163 studied HD patients were found positive for anti-HCV antibody by ELISA [27]. In a descriptive study conducted by Taziki, et al, they showed that the prevalence of HCV infection in HD patients has decreased significantly during the past decade in most HD units in the province of Mazandaran, northern Iran; in December 2001, the prevalence of antibody against HCV was 18%, whereas by December 2006, it was 12% [28]. The overall prevalence of anti-HCV antibodies was 12.7% in report of Ocak, et al [29]. Hussein, et al, studied 180 HD patients and identified 34 (18.9%) patients positive for anti-HCV antibody [30]. In our study, prevalence of HCV RNA was 50.8 % which was lower than other reports. In a study performed by Silva, et al, HCV-RNA was detected in 92 (%73.6%) of 125 anti-HCV-positive patients [26]. Dattolo, et al, showed that HCV-RNA was positive in 18 (75%) of 24 anti-HCV-positive subjects [31]. Mansour-Ghanaei, et al, showed that 10.42% of 163 HD patients were positive for HCV RNA by PCR [27]. Ocak, et al, reported a HCV RNA prevalence of 10.1% [29] The latter two prevalence rates which are lower than ours, were calculated based on all patients, whereas our rate was estimated based on anti-HCV-antibody-positive patients. In our study, significant correlation was found between anti-HCV antibody with gender and duration of HD. Furthermore, significant correlation was seen between HCV RNA seroprevalence and age and duration of HD.

Several studies were performed round the globe which focused on HCV seroprevalence in HD patients and its relationship with risk factors. These studies had variable results. In a study by Assarehzadegan, et al, the duration of treatment with HD was significantly associated with HBV- and HCV-positivity (p < 0.001) [23]. Sekkat, et al, reported a seroprevalence rate of HCV of 68.3%. HCV seropositivity was associated with longer duration of dialysis (p < 0.001) [32]. Soto-Salgado, et al, using bivariate analysis revealed that in HD patients age was significantly (p = 0.05) associated with anti-HCV positivity [33]. Salama, et al, I a multivariate analysis demonstrated that HCV status was linked to HD duration [34]. El-Amin, et al, found that prevalence of HCV was 23.7%; HCV seropositivity was associated with longer duration of dialysis (p < 0.001) and age of over 30 (p = 0.008) [35]. A high prevalence of HCV infection was observed in the study conducted by Iwasaki, et al; 23.9% and 26.8% of the 142 studied patients were positive for anti-HCV antibody and HCV RNA, respectively. These positive rates correlated with the duration of HD [36]. Freitas studied 163 patients in five dialysis units. The prevalence of anti-HCV was 11%. Length of HD was associated with HCV infection. HCV RNA was detected in 12 samples [37]. Jabbari, et al, studied 93 HD patients in Golestan province of Iran and found that the mean duration of HD was higher in anti-HCV-antibody positive cases (p < 0.05) [38]. Ohsawa, et al, reported that the prevalence of anti-HCV antibody was 12.5% in male HD patients and 8.5% in female HD patients [39]. Prevalence of HCV infection in Dialysis Unit Gracanica was 46.5%. Men were more HCV-positive (59.3%) (16 of 27 patients). In anti-HCV-positive patients, 85% were positive for HCV RNA. Duration of dialysis therapy was longer in anti-HCV-positive patients (6.8 years) than in anti-HCV-negative patients (1.6 years) [40]. In our study, seroprevalence of HBsAg was 1.4% in HD patients which was lower than that reported in other studies from our country and other regions. That would be attributed to effective vaccination and isolation of infected patients. Telaku, et al, reported a HBsAg prevalence rate of 12% [14]. Alavian, et al, reported that the seropositivity for HBsAg decreased from 3.8% in 1999 to 2.6% in 2006 [22]. Assarehzadegan, et al, reported that among 214 HD patients, 5.1% were positive for HBsAg [23]. In a study conducted by Boulaajaj, et al, on 186 chronic HD patients, the prevalence of HBV infection was 2% [24]. Mahdavizadeh, et al, found 64 (2.4%) HBsAg-positive patients in 2630 HD patients of Tehran province, Iran [41]. Al Hijazat, et al, showed that among 427 patients on HD, 5.9% became HBsAg-positive during the study period. Being on HD for longer than two years was more frequently noticed in the HBsAg-positive (88%) than in HBsAg-negative group (43%). Of these patients, 94% patients who remained HBsAg-negative and 4% of those who converted to HBsAg-positive were reportedly vaccinated [17]. There is low prevalence of HCV and HBV infection in HD population of our region and it can be decreased by HBV vaccination of ESRD patients before setting chronic HD, antiviral treatment and isolation of infected individuals.
